# ACE2 Receptor Usage across Animal Species by SARS-CoV-2 Variants

**DOI:** 10.3201/eid3108.241844

**Published:** 2025-08

**Authors:** Masato Hatta, Gloria P. Larson, Yasuko Hatta, Wei Wang, Nannan Jiang, Yu-Jin Jung, Li Wang, Xiaoyu Fan, Brenda M. Calderon, Gaston Bonenfant, Xudong Lin, Chenchen Feng, Dan Cui, Ginger Atteberry, Michael Currier, John Steel, David E. Wentworth, Bin Zhou

**Affiliations:** Centers for Disease Control and Prevention, Atlanta, Georgia, USA (M. Hatta, Y. Hatta, N. Jiang, L. Wang, X. Fan, B.M. Calderon, X. Lin, D. Cui, G. Atteberry, M. Currier, J. Steel, D.E. Wentworth, B. Zhou); Oak Ridge Institute for Science and Education, Oak Ridge, Tennessee, USA (G.P. Larson, G. Bonenfant, C. Feng); General Dynamics Information Technology, Inc., Falls Church, Virginia, USA (W. Wang, Y.-J. Jung)

**Keywords:** COVID-19, coronavirus disease, SARS-CoV-2, severe acute respiratory syndrome coronavirus 2, viruses, respiratory infections, ACE2, One Health, zoonoses

## Abstract

We analyzed the receptor-binding activity and infectivity of 6 representative SARS-CoV-2 lineages in cell lines expressing angiotensin-converting enzyme 2 proteins from 54 different animal species. All viruses demonstrated infectivity in a broad range of species. Susceptible animal species could serve as natural reservoirs or intermediate hosts for SARS-CoV-2.

SARS-CoV-2, the causative agent of COVID-19, has resulted in >775 million cases and 7 million deaths worldwide (*1*). Although the origin and the intermediate host(s) of this virus remain unclear, the virus has infected dozens of animal species presumably through reverse zoonosis, including wild animals such as white-tailed deer and companion animals such as cats and dogs (*2*–*5*). During 2020–2025, the virus has evolved rapidly, giving rise to thousands of variants; hundreds spread and were replaced by newer lineages. During that process, mutations accumulated in the SARS-CoV-2 genome, especially in the spike gene. For example, Omicron XBB.1.5 has acquired >40 nonsynonymous mutations in the spike gene compared with the wild-type index virus. Because the spike–receptor interaction is the initial and decisive step in coronavirus infection, amino acid changes in the spike protein can enhance or reduce infectivity in humans and other animal species, potentially altering the virus’s species specificity. Investigating spike interaction with a broad range of angiotensin-converting enzyme 2 (ACE2) receptors from different species is therefore crucial for understanding potential reservoirs before and after the virus’s emergence in humans and for enabling risk assessment of viruses that have crossed into new hosts through reverse zoonosis.

## The Study

**Figure 1:**
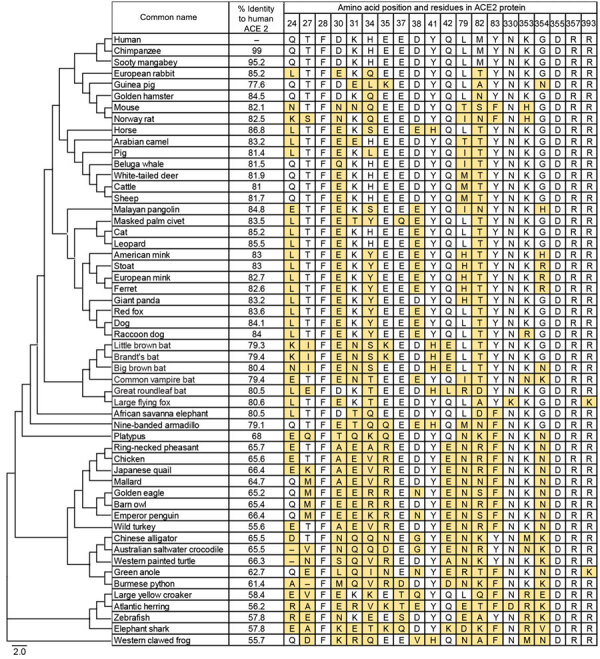
Sequence comparison of ACE2 proteins among 54 animal species with phylogenetic tree of ACE2 proteins in study of ACE2 receptor usage across animal species by SARS-CoV-2 variants. Protein sequence of ACE2 from various species are aligned at residues in the SARS-CoV-2 spike protein binding interface. Percent identity to human ACE2 was calculated by pairwise alignment of individual ACE2s to human ACE2. Residues differing from human ACE2 residues are highlighted in yellow. Scale bar indicates the number of amino acid substitutions based on ACE2 protein sequences. ACE2, angiotensin-converting enzyme 2.

We analyzed the ACE2 receptor activity of 54 animal species (36 mammals, 8 birds, 5 reptiles, 1 amphibian, and 4 fish) against various SARS-CoV-2 lineages that evolved over time (Figure 1; Appendix Table 1). Those animal species were selected to represent the broad diversity of ACE2 sequences and for other factors such as their potential role as reservoir of the SARS-CoV-2 progenitor (e.g., bats), potential role as intermediate host (e.g., pangolin, raccoon dog), close contact with humans (e.g., dog and cat), and indication of a large-scale reverse zoonotic event that appears to have led to enzootic disease (e.g., white-tailed deer). Compared with the human ACE2, the amino acid identity of the other 53 species ranged from 56% (wild turkey and western clawed frog) to 99% (chimpanzee) (Figure 1). We compared the sequences of the 20 ACE2 residues previously reported as critical in the spike–ACE2 binding interface across all 54 species (*6*,*7*).

**Figure 2:**
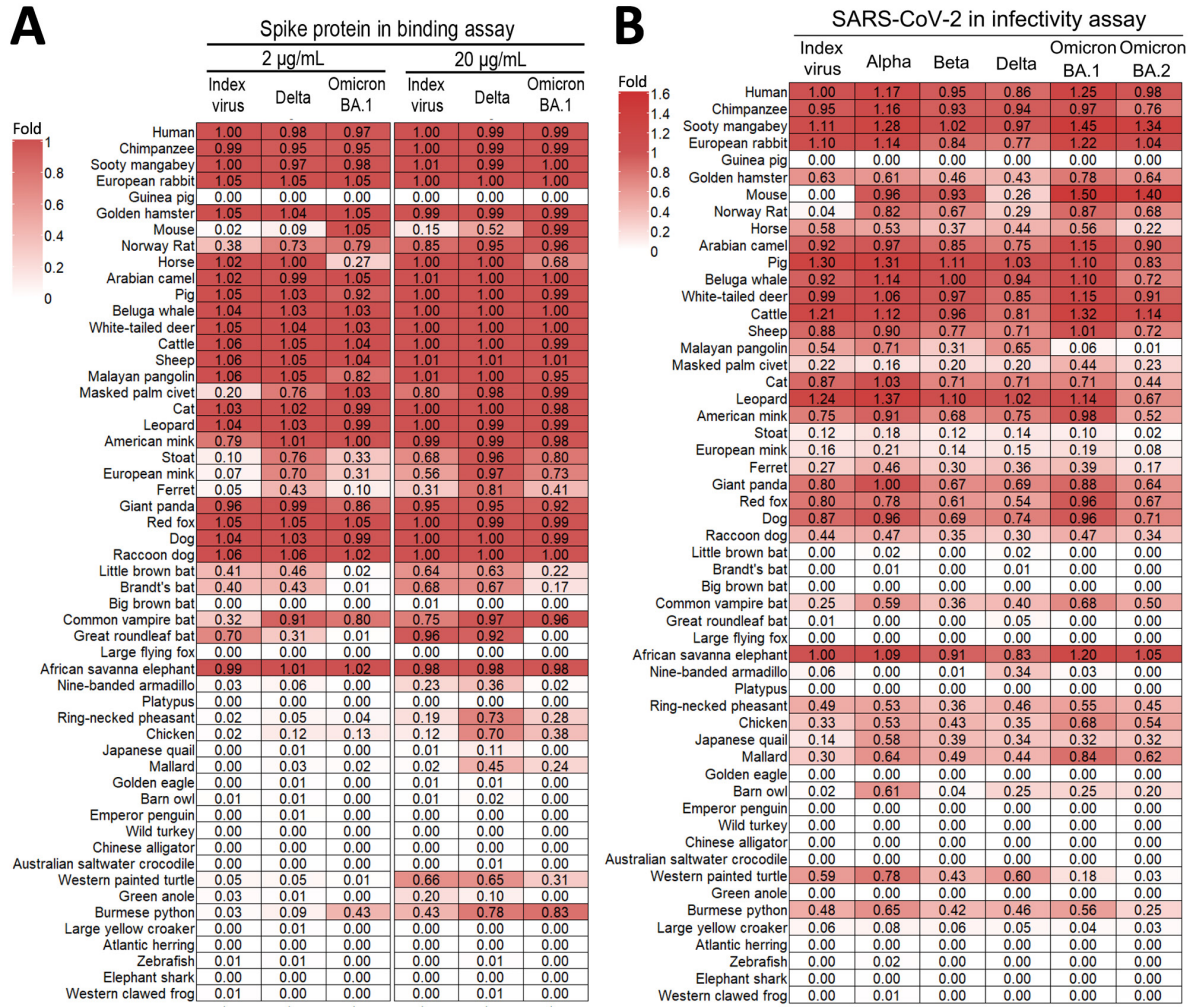
Heatmaps showing the binding strength of spike proteins (A) and infectivity of SARS-CoV-2 variants (B) to cells expressing ACE2 proteins from 54 animal species in study of ACE2 receptor usage across animal species by SARS-CoV-2 variants. The binding of the spike proteins to ACE2 was normalized to the reference group of index (wild-type) virus spike protein and human ACE2 cells (defined as 1) for both 2 µg/mL and 20 µg/mL spike protein concentrations. The representative data from 3 independent experiments are shown. The infectivity of SARS-CoV-2 reporter viruses was also normalized to the reference group of index virus and human ACE2 cells (defined as 1). The ratios, relative to the index virus and human ACE2 cells, are displayed as colors ranging from white to red. The experiment was performed in triplicate and the average was used in the heatmap. ACE2, angiotensin-converting enzyme 2.

To understand spike-ACE2 binding characteristics during the viral entry process, we developed 54 cell lines expressing different species of ACE2 proteins exogenously. We transfected a human ACE2 knockout HEK293T cell line (293T-ACE2-KO) with ACE2 expression plasmids. At 22–24 hours after transfection, we incubated the recombinant trimeric spike proteins of the SARS-CoV-2 index virus, the Delta variant, or the Omicron BA.1 variant with the ACE2-expressing cells. We analyzed spike protein binding by flow cytometry. Generally, increased binding was detected when the spike protein concentration increased from 2 µg/mL to 20 µg/mL (Appendix Figure 1). To compare the binding of the 3 spikes across ACE2 receptors, we normalized the flow cytometry signals to the index virus spike versus human ACE2 receptor reference group at each spike concentration (2 µg/mL and 20 µg/mL) (Figure 2, panel A). Overall, spike proteins bound efficiently to most of the mammalian ACE2s but showed little to no binding to ACE2s from birds, reptiles, amphibians, or fish. Specifically, none of the spike proteins bound to guinea pig ACE2, suggesting guinea pig is unlikely to be a susceptible animal model for SARS-CoV-2, which was recently confirmed (*8*). In contrast, all spike proteins bound efficiently to the ACE2 of golden hamster, which is widely used in SARS-CoV-2 studies. Although the spike protein from the index virus does not bind to mouse ACE2, the Delta spike protein gained ability to bind to mouse ACE2 at high concentration, and the Omicron spike protein bound to mouse ACE2 with efficiency comparable to human ACE2. In addition to those laboratory model animals, this assay illustrates that spike proteins can also bind to ACE2s of domesticated animals (such as rabbit, camel, pig, cattle, sheep, cat, and dog) and wild animals (such as whale, pangolin, leopard, panda, fox, raccoon dog, and elephant). Of note, compared with the index virus spike, the Delta and Omicron spikes showed increased binding to ACE2s of rat, palm civet, American mink, stoat, European mink, ferret, pheasant, chicken, mallard, and python but showed decreased binding to horse and turtle ACE2. The binding to bat ACE2s was variable depending on species. Those results indicate that the spike proteins of the index virus, Delta, and Omicron BA.1 have broad species specificity; however, differences in ACE2 binding specificity have emerged among SARS-CoV-2 variants.

Because viral entry goes beyond the spike–ACE2 binding step, we further explored ACE2 species specificity using infectious SARS-CoV-2 viruses, which require the ACE2 to be functional in mediating subsequent steps (e.g., fusion) of viral entry. We inoculated ACE2-transfected 293T-ACE2-KO cells with 10^4^ focus-forming units of GFP-expressing SARS-CoV-2 viruses possessing the spike gene from the wild-type virus or the Alpha, Beta, Delta, or Omicron BA.1 and BA.2 lineages (*9*). We counted GFP-positive cells at 20–24 hours after inoculation and expressed results as ratio to the wild-type virus spike protein versus human ACE2 reference group. All tested viruses exhibited broad species specificity for ACE2 proteins; variants demonstrated differential infectivity against certain ACE2 receptors, largely consistent with the results of the spike protein–ACE2 binding assay (Figure 2). Of note, Omicron lineage variants lost the ability to infect pangolin ACE2-expressing cells, and BA.2 showed lower infectivity for horse ACE2-expressing cells. The common vampire bat is the only species that showed susceptibility among the 6 bat species analyzed, both in the spike–ACE2 binding assay and the live-virus infectivity assay. Little brown bat and Brandt’s bat were moderately positive in the binding assay but not in the infectivity assay, supporting the value of performing the infectivity assay. The successful infection of cells expressing turtle and python ACE2 is also intriguing. Chicken and quail have been demonstrated to be nonsusceptible to SARS-CoV-2 infection (*10*). However, in this study, the cells expressing ACE2 of those species were susceptible to SARS-CoV-2 infection, although the infectivity was not high. Additional host factors, such as the distribution and amount of ACE2 proteins in tissues, cellular proteins involved in viral replication, or innate immunity, would affect the establishment of infection in animals exposed to SARS-CoV-2. For species of particular interest, further investigation through animal infection experiments is necessary to confirm susceptibility.

## Conclusions

The susceptibility of animal species to SARS-CoV-2 has been diligently studied in various in silico, in vitro, in vivo, and epidemiologic analyses since the pandemic began (Appendix Table 2). However, the differences in ACE2 specificity among SARS-CoV-2 variants, especially Omicron lineages, have not been comprehensively studied. In this study, we demonstrated the wide range of species specificity of SARS‐CoV‐2 variants and the differences in their ability to use various ACE2 proteins as receptors. The dozens of amino acid differences in the spike proteins could affect the variants’ pathogenicity, antigenicity, transmissibility, infectivity, and host species specificity. Further structural or mutagenesis analysis of the spike proteins and the ACE2 proteins could identify the key interacting amino acids (Figure 1) responsible for species specificity. This study suggests that susceptible animal species could evolutionarily serve as natural reservoirs or intermediate hosts, transmitting SARS-CoV-2 to other species or back to humans, potentially leading to future outbreaks or a new pandemic driven by novel SARS-CoV-2 variants with animal-adapted mutations.

Appendix Additional information about ACE2 receptor usage across animal species by SARS-CoV-2 variants

## References

[1] World Health Organization. WHO coronavirus (COVID-19) dashboard [cited 2024 Aug 6]. https://covid19.who.int

[2] Doliff R, Martens P. Cats and SARS-CoV-2: a scoping review. Animals (Basel). 2022;12:1413. 10.3390/ani12111413 35681877 PMC9179433

[3] Liew AY, Carpenter A, Moore TA, Wallace RM, Hamer SA, Hamer GL, et al.; Companion Animals Working Group. Clinical and epidemiologic features of SARS-CoV-2 in dogs and cats compiled through national surveillance in the United States. J Am Vet Med Assoc. 2023;261:480–9. 10.2460/javma.22.08.0375 36595371 PMC10038921

[4] Goldberg AR, Langwig KE, Brown KL, Marano JM, Rai P, King KM, et al. Widespread exposure to SARS-CoV-2 in wildlife communities. Nat Commun. 2024;15:6210. 10.1038/s41467-024-49891-w 39075057 PMC11286844

[5] Pandit R, Matthews QLAA. SARS-CoV-2: companion animal transmission and variants classification. Pathogens. 2023;12:775. 10.3390/pathogens12060775 37375465 PMC10302237

[6] Lan J, Ge J, Yu J, Shan S, Zhou H, Fan S, et al. Structure of the SARS-CoV-2 spike receptor-binding domain bound to the ACE2 receptor. Nature. 2020;581:215–20. 10.1038/s41586-020-2180-5 32225176

[7] Shang J, Ye G, Shi K, Wan Y, Luo C, Aihara H, et al. Structural basis of receptor recognition by SARS-CoV-2. Nature. 2020;581:221–4. 10.1038/s41586-020-2179-y 32225175 PMC7328981

[8] Iwatsuki-Horimoto K, Kiso M, Ito M, Yamayoshi S, Kawaoka Y. Sensitivity of rodents to SARS-CoV-2: gerbils are susceptible to SARS-CoV-2, but guinea pigs are not. Npj Viruses. 2024;2:59. 10.1038/s44298-024-00068-8 PMC11721077 40295803

[9] Wang L, Kainulainen MH, Jiang N, Di H, Bonenfant G, Mills L, et al.; SSEV Bioinformatics Working Group. Differential neutralization and inhibition of SARS-CoV-2 variants by antibodies elicited by COVID-19 mRNA vaccines. Nat Commun. 2022;13:4350. 10.1038/s41467-022-31929-6 35896523 PMC9328008

[10] Suarez DL, Pantin-Jackwood MJ, Swayne DE, Lee SA, DeBlois SM, Spackman E. Lack of susceptibility to SARS-CoV-2 and MERS-CoV in poultry. Emerg Infect Dis. 2020;26:3074–6. 10.3201/eid2612.202989 33219803 PMC7706925

