# Supplementary material for: ACE2 Receptor Usage across Animal Species by SARS-CoV-2 Variants
Source: Emerg Infect Dis. 2025 Aug;31(8):1640–4. doi: 10.3201/eid3108.241844 (PMC12309770; doi:10.3201/eid3108.241844)
Supplement: Appendix — Additional information about ACE2 receptor usage across animal species by SARS-CoV-2 variants [file 24-1844-Techapp-s1.pdf]

Article DOI: https://doi.org/10.3201/eid3108.241844

EID cannot ensure accessibility for supplementary materials supplied by authors. Readers who have difficulty accessing supplementary content should contact the authors for assistance.

ACE2 Receptor Usage Across Animal Species by SARS-CoV-2 Variants

Appendix

Materials and Methods

Biosafety Statement

All work involving infectious SARS-CoV-2 virus, including recombinant reporter virus, was performed in CDC Biosafety Level 3 facilities with enhanced practices (BSL-3E). All personnel working with the virus were trained with relevant safety and procedure-specific protocols, and their competency for performing the work in the BSL-3E laboratories was certified. Recombinant DNA work was approved by CDC's Institutional Biosafety Committee (IBC). For sequencing, virus was inactivated following protocols approved by CDC's Laboratory Safety Review Board (LSRB) with a witness confirming that all steps were performed correctly to ensure complete inactivation of virus. After receiving appropriate approvals, inactivated virus was transferred to BSL-2E laboratories for downstream processing.

Sequence Alignment and Phylogeny

ACE2 protein sequences of 54 animal species were obtained from either UniProt (https://www.uniprot.org/) or NCBI database (https://www.ncbi.nlm.nih.gov/protein/) (Appendix Table 1). The phylogenetic tree was constructed using the Jukes-Cantor genetic distance model and neighbor-joining method in Geneious Prime (https://www.geneious.com/prime/) and visualized/explored in FigTree v1.4.4 (http://tree.bio.ed.ac.uk/software/figtree/). Protein sequence alignment of ACE2s at residues in the SARS-CoV-2 spike protein binding interface were performed by individually pairwise alignment to human ACE2 (hACE2) using MUSCLE

alignment in Geneious (version 2019.2.3, https://www.geneious.com/). Percent identity and residues numbers use hACE2 as a reference.

Cells

SARS-CoV-2 nucleocapsid protein expressing cell line (VeroE6-N) (*1*) and VeroE6/TMPRSS2 cells (JCRB Cell Bank, JCRB1819) (*2*) were maintained in DMEM supplemented with 10% FBS and 0.2 mg/ml geneticin. 293T-ACE2 knockout (293T-ACE2 KO) cells (CSC-RT2688, clone #34) (Creative Biogene, https://www.creative-biogene.com/) were grown in DMEM supplemented with 10% FBS. All cell lines were cultured at 37°C in a 5% CO_2 incubator.

Generation of SARS-CoV-2 Reporter Viruses

Recombinant SARS-CoV-2 viruses with a mNeonGreen fluorescent reporter gene were generated by reverse genetics as described previously (*1*). Briefly, the DNA clone for SARS-CoV-2 index virus (Wuhan-Hu-1, GenBank Accession Number: NC_045512.2) flanked by a T7 promoter sequence at the 5′ end and a linearization site at the 3′ end in a bacterial artificial chromosome (BAC) vector was used. The ORF7a gene of the DNA clone was replaced with a human codon-optimized mNeonGreen gene. For generating the DNA clones of descendent SARS-CoV-2 variants (Alpha, Beta, Delta, and Omicron BA.1 and BA.2), the spike gene of index virus in BAC vector was excised and replaced with synthetic spike genes of the variants. The sequence of the obtained DNAs was verified by Illumina next-generation sequencing (NGS) (Illumina, https://www.illumina.com/). VeroE6-N cells were then transfected with in vitro transcribed RNA from these plasmids by an electroporation with the Nucleofector Kit V (Lonza, https://www.lonza.com/). At 18 to 24 hours post-transfection, the supernatants were collected and amplified in VeroE6/TMPRSS2 cells. The amplified viruses were titrated by the focus forming unit (FFU) assay described previously (*1*) and sequenced by NGS to confirm no unwanted mutations in the spike gene.

ACE2 plasmids

Full-length ACE2 genes of 54 animal species obtained from NCBI GenBank. The ACE2 cDNAs of golden hamster, racoon dog, masked palm civet, stoat, European mink, and Australian saltwater crocodile were codon optimized and that of guinea pig was a chimeric construct with the sequence modified due to missing information for certain residues. The ACE2s were

generated and cloned into the pcDNA3.1(+)-P2A-eGFP vector or pCMV-IRES-mCherry2 vector (*3*) by GenScript cloning services (GenScript, https://www.genscript.com/) or in our laboratory. ACE2 proteins were expressed in transfected cells as a precursor protein with enhanced GFP (eGFP) and separated by a self-cleaving P2A sequence or as ACE2 protein with a 6X histidine tag at C-terminus and mCherry2 fluorescent protein expressed through internal ribosome entry site (IRES) in transfected cells.

Spike-ACE2 Binding Assay by Flow Cytometry

293T-ACE2 KO cells were transfected with ACE2 expression plasmids and harvested 22 to 24 hours post-transfection. Cells were incubated with 2 μg/mL or 20 μg/mL 6X histidine-tagged SARS-CoV-2 spike protein on ice for 15–30 minutes. The spike trimer of the index virus was prepared as described previously (*3*) and those of Delta and Omicron BA.1 variants were obtained from Sino Biological (https://www.sinobiological.com/)]. Cells were then washed and incubated with anti-His-Alexa Fluor 647 antibody (clone 4E3D10H2/E3) (Thermo Fisher Scientific, https://www.thermofisher.com/) on ice for 15–30 minutes, washed, fixed with formalin, and measured by FACSCanto2. Flow cytometry data were analyzed using FlowJo software (https://www.flowjo.com/). Single cell populations were selected by FSC vs SSC. eGFP positive cells were gated and the mean fluorescence intensity (MFI) of Alexa Fluor 647 and % Alexa Fluor 647 positive cell population were calculated. The heatmap was created using the Complex package v2.13.1 in RStudio version 1.4.1717. The transfection efficiency and eGFP expression levels in transfected 293T-ACE2 KO cells were confirmed to be similar among the cells expressing species-specific ACE2s (Appendix Figure 2A and 2B).

In Vitro Susceptibility Assay with Reporter Virus

293T-ACE2 KO cells in 96 well plates (3,000 cells per well) were transfected with ACE2 expression plasmids. TMPRSS2-OFPSpark (HG13070-ACR) (SinoBiological, https://www.sinobiological.com/) plasmid was co-transfected to support efficient SARS-CoV-2 infection (*4*,*5*). Twenty to 24 hours after transfection, the transfection efficiency was analyzed via mCherry2 expression using the BioTek Cytation 7 Cell Imaging Multi-Mode Reader (Cytation 7) (Agilent, https://www.agilent.com/) and was confirmed to be similar among the transfected cells (Appendix Figure 3A). Transfected cells were also imaged with a Keyence fluorescence microscope (BZ-X810) (Keyence, https://www.keyence.com) or Cytation 7 (Appendix Figure 3B). The transfected cells were then infected with SARS-CoV-2 reporter

viruses. Twenty to 24 hours after infection, the number of the cells expressing mNeonGreen protein was counted using the Cytation 7. The infectivity of SARS-CoV-2 reporter viruses was normalized to the infectivity of the index virus in cells overexpressing hACE2, defined as 1. The fold changes of infectivity to that of hACE2 were displayed with the heatmap created by the Complex package v2.13.1 in RStudio version 1.4.1717.

To determine the proper virus dose to use for infection, we transfected 293T-ACE2 KO cells with 32 ng of hACE2 plasmid and infected with various amount of Omicron BA.2 variant reporter virus (0.5 × 10^4, 10^4, and 2 × 10^4 FFU per well). The results demonstrated a similar infectivity among these 3 infectious doses (Appendix Figure 4). We used 10^4 FFU per well of infectious dose in the main experiments.

References

1. Wang L, Kainulainen MH, Jiang N, Di H, Bonenfant G, Mills L, et al.; SSEV Bioinformatics Working Group. Differential neutralization and inhibition of SARS-CoV-2 variants by antibodies elicited by COVID-19 mRNA vaccines. Nat Commun. 2022;13:4350. PubMed https://doi.org/10.1038/s41467-022-31929-6

2. Matsuyama S, Nao N, Shirato K, Kawase M, Saito S, Takayama I, et al. Enhanced isolation of SARS-CoV-2 by TMPRSS2-expressing cells. Proc Natl Acad Sci U S A. 2020;117:7001–3. PubMed https://doi.org/10.1073/pnas.2002589117

3. Wang L, Fan X, Bonenfant G, Cui D, Hossain J, Jiang N, et al. Susceptibility to SARS-CoV-2 of Cell Lines and Substrates Commonly Used to Diagnose and Isolate Influenza and Other Viruses. Emerg Infect Dis. 2021;27:1380–92. PubMed https://doi.org/10.3201/eid2705.210023

4. Hoffmann M, Kleine-Weber H, Schroeder S, Krüger N, Herrler T, Erichsen S, et al. SARS-CoV-2 Cell Entry Depends on ACE2 and TMPRSS2 and Is Blocked by a Clinically Proven Protease Inhibitor. Cell. 2020;181:271–280.e8. PubMed https://doi.org/10.1016/j.cell.2020.02.052

5. Ou T, Mou H, Zhang L, Ojha A, Choe H, Farzan M. Hydroxychloroquine-mediated inhibition of SARS-CoV-2 entry is attenuated by TMPRSS2. PLoS Pathog. 2021;17:e1009212. PubMed https://doi.org/10.1371/journal.ppat.1009212

Appendix Table 1. The animal species used in this study and the accession numbers of their ACE2 protein sequences

Common Name	Scientific Name	NCBI Accession
human	*Homo sapiens*	NP_001358344.1
chimpanzee	*Pan troglodytes*	XP_016798468.1
sooty mangabey	*Cercocebus atys*	XP_011891201.1
European rabbit	*Oryctolagus cuniculus*	XP_002719891.1
guinea pig	*Cavia porcellus*	XP_023417808.1*
golden hamster	*Mesocricetus auratus*	XP_005074266.1
mouse	*Mus musculus*	NP_001123985.1
Norway rat	*Rattus norvegicus*	NP_001012006.1
horse	*Equus caballus*	XP_001490241.1
Arabian camel	*Camelus dromedarius*	XP_010991717.1
pig	*Sus scrofa*	NP_001116542.1
beluga whale	*Delphinapterus leucas*	XP_022418360.1
white-tailed deer	*Odocoileus virginianus*	XP_070318733
Cattle	*Bos taurus*	NP_001019673.2
Sheep	*Ovis aries*	XP_011961657.1
Malayan pangolin	*Manis javanica*	XP_017505752.1
masked palm civet	*Paguma larvata*	AAX63775.1
cat	*Felis catus*	NP_001034545.1
leopard	*Panthera pardus*	XP_019273508.1
American mink	*Neogale vison*	QPL12211.1
stoat	*Mustela erminea*	XP_032187679.1
European mink	*Mustela lutreola*	QNC68911.1
Ferret	*Mustela putorius furo*	NP_001297119.1
giant panda	*Ailuropoda melanoleuca*	XP_002930657.1
red fox	*Vulpes vulpes*	XP_025842512.1
dog	*Canis lupus familiaris*	XP_005641049.1
raccoon dog	*Nyctereutes procyonoides*	ABW16956.1
little brown bat	*Myotis lucifugus*	XP_006088637.1
Brandt's bat	*Myotis brandtii*	XP_014399780.1
big brown bat	*Eptesicus fuscus*	XP_008153150.1
common vampire bat	*Desmodus rotundus*	XP_024425698.1
great roundleaf bat	*Hipposideros armiger*	XP_019522936.1
large flying fox	*Pteropus vampyrus*	XP_011361275.1
African savanna elephant	*Loxodonta africana*	XP_003416023.1
nine-banded armadillo	*Dasypus novemcinctus*	XP_004449124.1
platypus	*Ornithorhynchus anatinus*	XP_001515597.2
ring-necked pheasant	*Phasianus colchicus*	XP_031451919.1
chicken	*Gallus gallus*	XP_416822.2
Japanese quail	*Coturnix japonica*	XP_015742063.1
mallard	*Anas platyrhynchos*	XP_012949915.2
golden eagle	*Aquila chrysaetos chrysaetos*	XP_029855025.1
barn owl	*Tyto alba*	XP_032865981.1
emperor penguin	*Aptenodytes forsteri*	XP_009275140.1
wild turkey	*Meleagris gallopavo*	XP_019467554.1
Chinese alligator	*Alligator sinensis*	XP_025066628.1
Australian saltwater crocodile	*Crocodylus porosus*	XP_019384826.1
western painted turtle	*Chrysemys picta bellii*	XP_005287845.1
green anole	*Anolis carolinensis*	XP_008105455.1
Burmese python	*Python bivittatus*	XP_007431942.2
large yellow croaker	*Larimichthys crocea*	XP_010730146.1
Atlantic herring	*Clupea harengus*	XP_031414786.1
zebrafish	*Danio rerio*	NP_001007298.1
elephant shark	*Callorhinchus milii*	XP_007889845.1
western clawed frog	*Xenopus tropicalis*	XP_002938293.2

*The guinea pig ACE2 sequence was updated in 2024 with NCBI reference sequence XP_063099000

Appendix Table 2. In silico, in vitro, in vivo, and epidemiologic analyses of the susceptibility of animal species to SARS-CoV-2

Study listing
Audino T, Berrone E, Grattarola C, Giorda F, Mattioda V, Martelli W, et al. Potential SARS-CoV-2 Susceptibility of Cetaceans Stranded along the Italian Coastline. Pathogens. 2022 Sep 25;11 (10).
Bouricha EM, Hakmi M, Akachar J, Belyamani L, Ibrahimi A. In silico analysis of ACE2 orthologs to predict animal host range with high susceptibility to SARS-CoV-2. 3 Biotech. 2020 Nov;10(11):483.
Damas J, Hughes GM, Keough KC, Painter CA, Persky NS, Corbo M, et al. Broad host range of SARS-CoV-2 predicted by comparative and structural analysis of ACE2 in vertebrates. Proc Natl Acad Sci U S A. 2020 Sep 8;117(36):22311–22.
Delgado Blanco J, Hernandez-Alias X, Cianferoni D, Serrano L. In silico mutagenesis of human ACE2 with S protein and translational efficiency explain SARS-CoV-2 infectivity in different species. PLoS Comput Biol. 2020 Dec;16(12):e1008450.
Farber I, Kruger J, Rocha C, Armando F, von Kockritz-Blickwede M, Pohlmann S, et al. Investigations on SARS-CoV-2 Susceptibility of Domestic and Wild Animals Using Primary Cell Culture Models Derived from the Upper and Lower Respiratory Tract. Viruses. 2022 Apr 16;14 (4).
Hossain MG, Javed A, Akter S, Saha S. SARS-CoV-2 host diversity: An update of natural infections and experimental evidence. J Microbiol Immunol Infect. 2021 Apr;54 (2):175–81.
Kaushik R, Kumar N, Zhang KYJ, Srivastava P, Bhatia S, Malik YS. A novel structure-based approach for identification of vertebrate susceptibility to SARS-CoV-2: Implications for future surveillance programmes. Environ Res. 2022 Sep;212(Pt C):113303.
Kondo T, Suzuki R, Yajima H, Kawahara S, Yamaya K, Ichikawa T, et al. Determinants of susceptibility to SARS-CoV-2 infection in murine ACE2. J Virol. 2025 May 12:e0054325.
Kruglikov A, Rakesh M, Wei Y, Xia X. Applications of Protein Secondary Structure Algorithms in SARS-CoV-2 Research. J Proteome Res. 2021 Mar 5;20 (3):1457–63.
Lam SD, Bordin N, Waman VP, Scholes HM, Ashford P, Sen N, et al. SARS-CoV-2 spike protein predicted to form complexes with host receptor protein orthologs from a broad range of mammals. Sci Rep. 2020 Oct 5;10 (1):16471.
Li L, Han P, Huang B, Xie Y, Li W, Zhang D, et al. Broader-species receptor binding and structural bases of Omicron SARS-CoV-2 to both mouse and palm-civet ACE2s. Cell Discov. 2022 Jul 12;8 (1):65.
Li P, Guo R, Liu Y, Zhang Y, Hu J, Ou X, et al. The Rhinolophus affinis bat ACE2 and multiple animal orthologs are functional receptors for bat coronavirus RaTG13 and SARS-CoV-2. Sci Bull (Beijing). 2021 Jun 30;66(12):1215–27.
Li S, Yang R, Zhang D, Han P, Xu Z, Chen Q, et al. Cross-species recognition and molecular basis of SARS-CoV-2 and SARS-CoV binding to ACE2s of marine animals. Natl Sci Rev. 2022 Sep;9 (9):nwac122.
Liu Y, Hu G, Wang Y, Ren W, Zhao X, Ji F, et al. Functional and genetic analysis of viral receptor ACE2 orthologs reveals a broad potential host range of SARS-CoV-2. Proc Natl Acad Sci U S A. 2021 Mar 23;118(12).
Luan J, Lu Y, Jin X, Zhang L. Spike protein recognition of mammalian ACE2 predicts the host range and an optimized ACE2 for SARS-CoV-2 infection. Biochem Biophys Res Commun. 2020 May 21;526 (1):165–9.
Ma C, Gong C. ACE2 models of frequently contacted animals provide clues of their SARS-CoV-2 S protein affinity and viral susceptibility. J Med Virol. 2021 Jul;93 (7):4469–79.
Pach S, Nguyen TN, Trimpert J, Kunec D, Osterrieder N, Wolber G. ACE2-Variants Indicate Potential SARS-CoV-2-Susceptibility in Animals: A Molecular Dynamics Study. Mol Inform. 2021 Sep;40 (9):e2100031.
Praharaj MR, Garg P, Kesarwani V, Topno NA, Khan RIN, Sharma S, et al. SARS-CoV-2 Spike Glycoprotein and ACE2 Interaction Reveals Modulation of Viral Entry in Wild and Domestic Animals. Front Med (Lausanne). 2021;8:775572.
Rutherford C, Kafle P, Soos C, Epp T, Bradford L, Jenkins E. Investigating SARS-CoV-2 Susceptibility in Animal Species: A Scoping Review. Environ Health Insights. 2022;16:11786302221107786.
Shi J, Wen Z, Zhong G, Yang H, Wang C, Huang B, et al. Susceptibility of ferrets, cats, dogs, and other domesticated animals to SARS-coronavirus 2. Science. 2020 May 29;368(6494):1016–20.
Wei Y, Aris P, Farookhi H, Xia X. Predicting mammalian species at risk of being infected by SARS-CoV-2 from an ACE2 perspective. Sci Rep. 2021 Jan 18;11 (1):1702.
Zhai X, Sun J, Yan Z, Zhang J, Zhao J, Zhao Z, et al. Comparison of Severe Acute Respiratory Syndrome Coronavirus 2 Spike Protein Binding to ACE2 Receptors from Human, Pets, Farm Animals, and Putative Intermediate Hosts. J Virol. 2020 Jul 16;94(15).
Zhang Y, Wei M, Wu Y, Wang J, Hong Y, Huang Y, et al. Cross-species tropism and antigenic landscapes of circulating SARS-CoV-2 variants. Cell Rep. 2022 Mar 22;38(12):110558.
Zhao J, Cui W, Tian BP. The Potential Intermediate Hosts for SARS-CoV-2. Front Microbiol. 2020;11:580137.
Zhao X, Chen D, Szabla R, Zheng M, Li G, Du P, et al. Broad and Differential Animal Angiotensin-Converting Enzyme 2 Receptor Usage by SARS-CoV-2. J Virol. 2020 Aug 31;94(18).

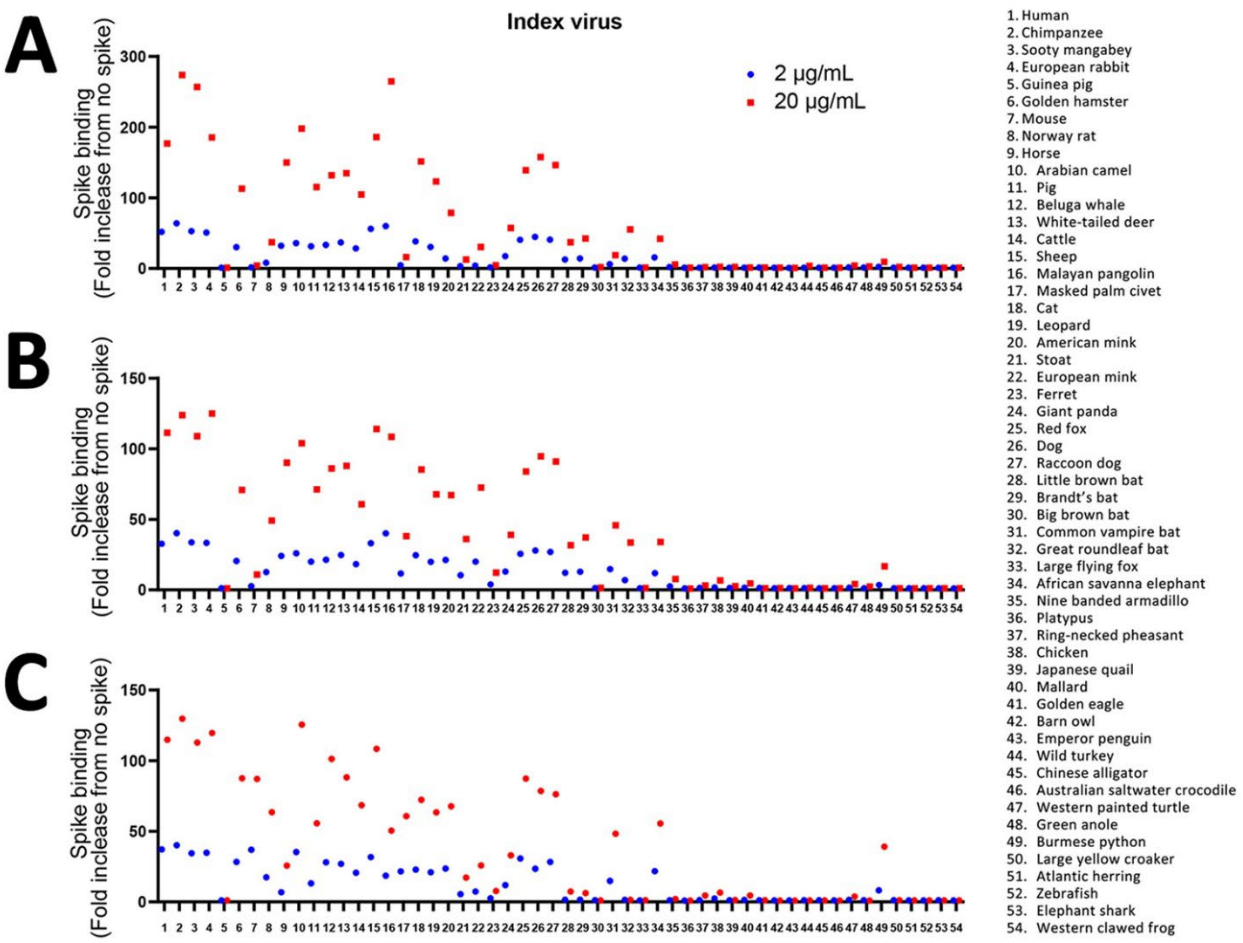

Appendix Figure 1. Spike-ACE2 Binding in flow cytometry binding assay. 293T-ACE2 KO cells were transfected with ACE2 expression plasmids and incubated with 2 μg/mL or 20 μg/mL of histidine-tagged SARS-CoV-2 spike proteins. Cells were then washed and incubated with anti-His-Alexa Fluor 647 antibody and measured by FACSCanto2. The mean fluorescence intensity (MFI) of Alexa Fluor 647 were calculated and plotted as fold increase from mock treated control cells. The representative data from 3 independent experiments are shown.

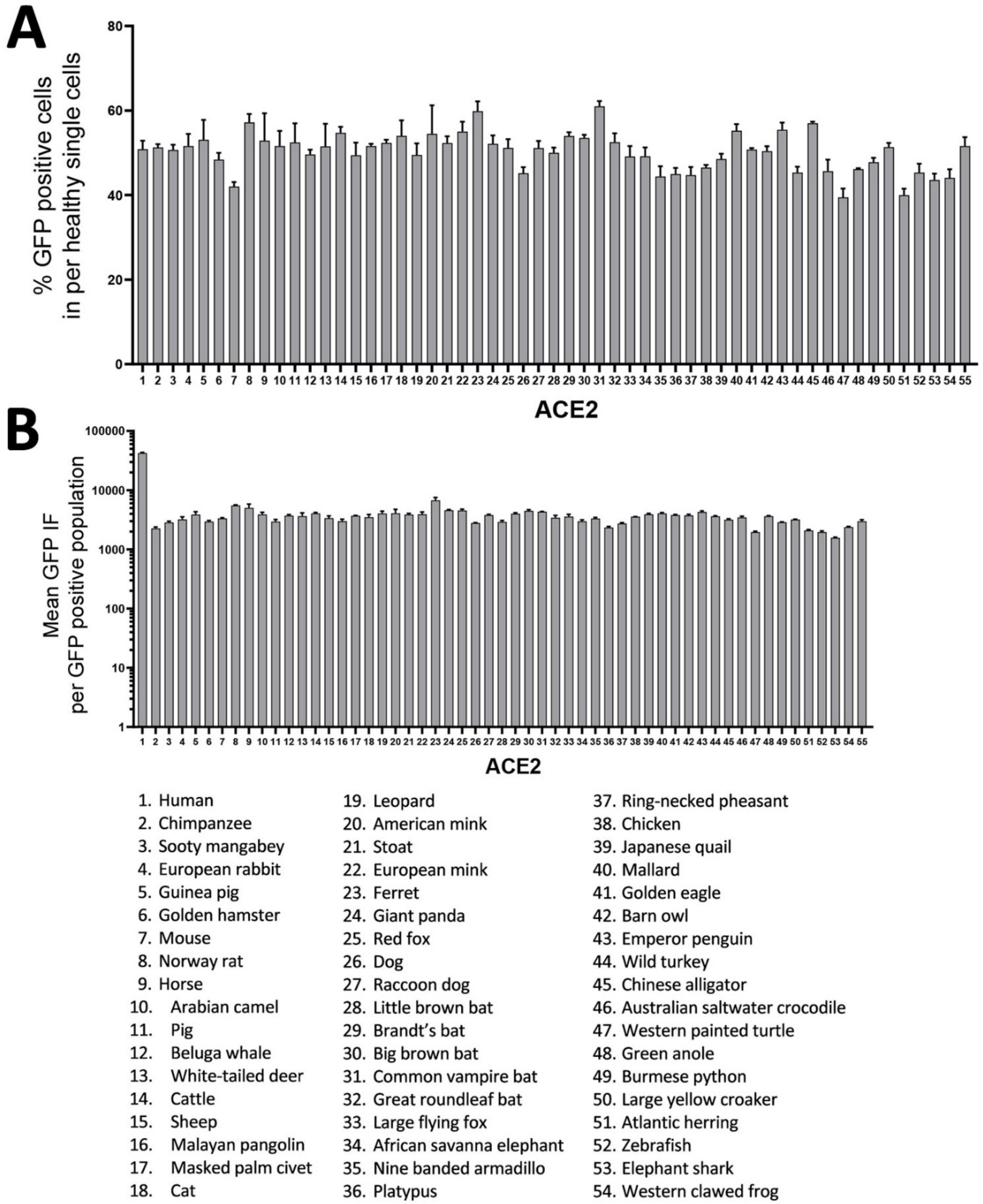

Appendix Figure 2. The transfection efficiency (A) and eGFP expression levels (B) in transfected 293T-ACE2 KO cells in the spike-ACE2 binding assay. The efficiency was calculated as the percent of eGFP positive cells in the total healthy population of transfected cells in the well. eGFP expression levels were determined by measuring the eGFP mean fluorescence intensity (MFI) signal of all eGFP positive cells. The representative data from 4 independent experiments are shown.

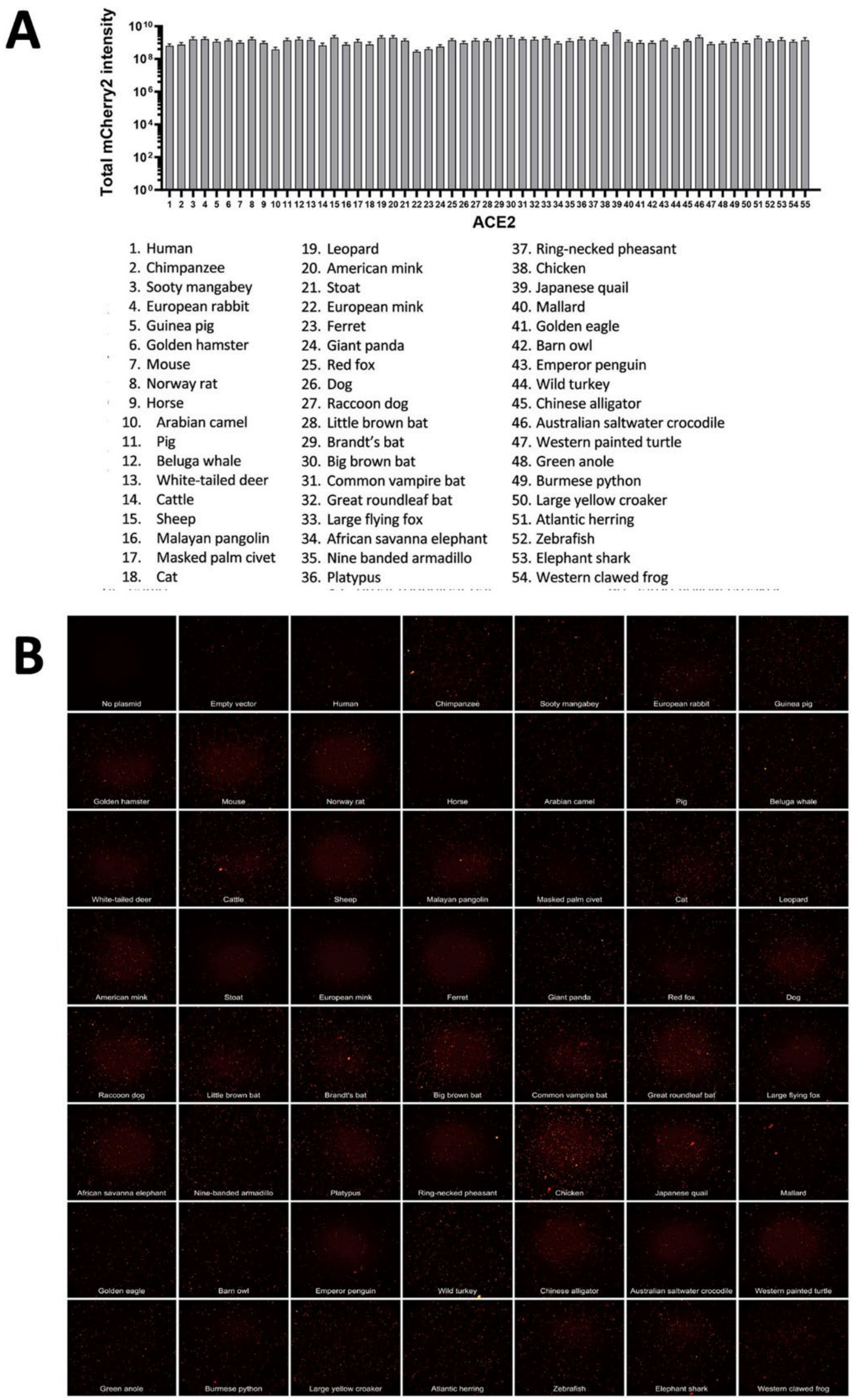

Appendix Figure 3. The total fluorescence intensity of mCherry2 proteins expressed in transfected 293T-ACE2 KO (A) and the mCherry2 fluorescence images of transfected 293T-ACE2 KO cells (B) in in vitro susceptibility assay on day 1 post-transfection. The data were obtained from 13 replicates (A). The representative images from 3 independent experiments are shown (B).

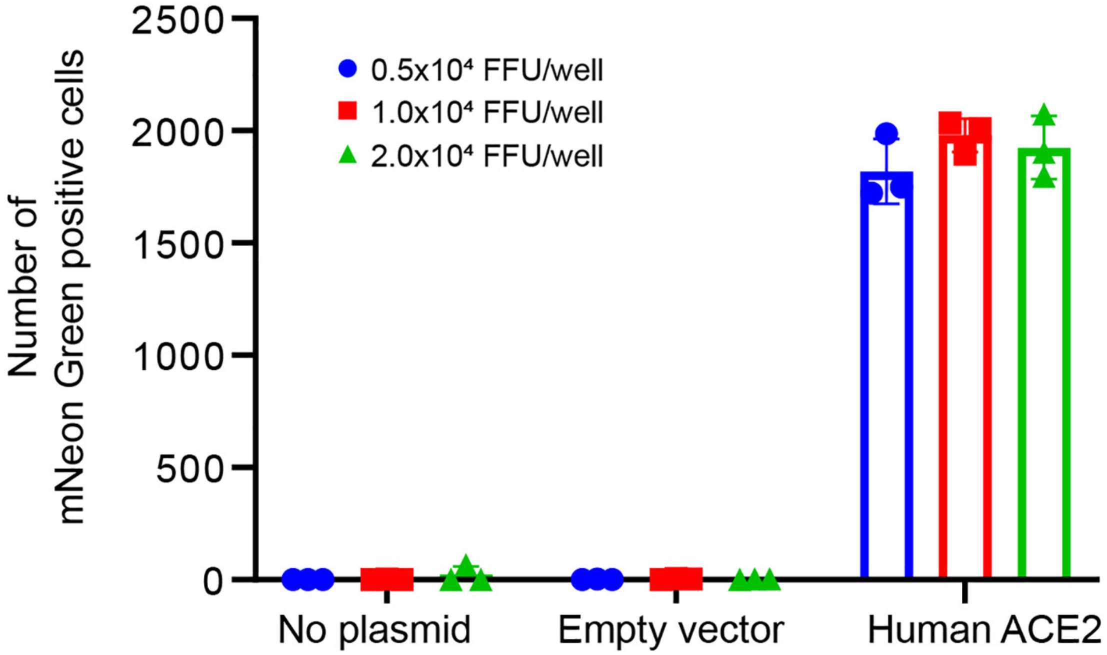

Appendix Figure 4. Determination of the virus dose used for infection in the in vitro SARS-CoV-2 susceptibility assay. 293T-ACE2 KO cells transfected with 32 ng of hACE2 expression plasmid were infected with various doses of the Omicron BA.2 reporter virus. The number of reporter virus-infected cells (mNeonGreen positive cells) was counted by Cytation 7. The experiment was performed in triplicate.
